# Effects of Dietary Brazilian Palm Oil (*Mauritia flexuosa* L.) on Cholesterol Profile and Vitamin A and E Status of Rats

**DOI:** 10.3390/molecules20059054

**Published:** 2015-05-19

**Authors:** Jailane de Souza Aquino, Juliana Késsia Barbosa Soares, Marciane Magnani, Thayza Christina Montenegro Stamford, Robson de Jesus Mascarenhas, Renata Leite Tavares, Tânia Lúcia Montenegro Stamford

**Affiliations:** 1 Departamento de Nutrição/CCS, Universidade Federal da Paraíba, Campus I, s/n. Castelo Branco, 58051-900 João Pessoa-PB, Brasil; E-Mail: renataltav@gmail.com; 2 Departamento de Nutrição/CES, Universidade Federal de Campina Grande, Olho d’agua da Bica, s/n, 58175-000 Cuité-PB, Brasil; E-Mail: julianakessia2@gmail.com; 3 Departamento de Engenharia de Alimentos, Universidade Federal da Paraíba, Campus I, s/n. Castelo Branco, 58051-970 João Pessoa-PB, Brasil; E-Mail: magnani2@gmail.com; 4 Departamento de Medicina Tropical/CCS, Universidade Federal de Pernambuco, Campus Recife, s/n, Cidade Universitária, 50670-901 Recife-PE, Brasil; E-Mail: thayzastamford@yahoo.com.br; 5 Instituto Federal de Educação, Ciência e Tecnologia do Sertão Pernambucano, Campus Petrolina, BR 407, km 08, Jd. São Paulo, 56314-520 Petrolina-PE, Brasil; E-Mail: robsonjmjm@gmail.com; 6 Departamento de Nutrição/ CCS, Universidade Federal de Pernambuco, Campus Recife, s/n, Cidade Universitária, 50670-901 Recife-PE, Brasil; E-Mail: tlmstamford@yahoo.com.br

**Keywords:** α-tocopherol, lipids, refined oil, retinol

## Abstract

*In vitro* studies have been carried out to establish the nutritional differences between crude and refined vegetable oils; however, the impact of the consumption of these foods on metabolism, in particular the effect of buriti oil, needs to be further evaluated. The aim of this study was to evaluate the biochemical and murine parameters and the vitamin A and E status in young rats fed with diets supplemented with crude or refined buriti oil. The animals (n = 30) were randomized into three groups receiving diet added of soybean oil (control), crude buriti oil (CBO) and refined buriti oil (RBO) for 28 days. Rats fed with diet added of refined buriti oil (RBO) showed reduced total cholesterol (up to 60.27%), LDL (64.75%), triglycerides (55.47%) and enzyme aspartate transaminase (21.57%) compared to those fed with diet added of crude oil. Serum and hepatic retinol and tocopherol were higher by two to three times in CBO and RBO groups compared to the control group, but no differences were observed for murine parameters. The results indicate that buriti oil is an important source of the antioxidant vitamins A and E, and refined buriti oil is suggested as alternative to improve the lipid profile of healthy rats.

## 1. Introduction

The compositions of crude oils obtained from plant species contain many bioactive compounds such as essential fatty acids and antioxidant vitamins. Studies involving diets with added vegetable oils have reported significant beneficial effects on health, particularly on the vitamin status and the lipid-lowering effects on humans, which is directly associated with the modulation of the antioxidant system homeostasis [1] and reduction of adipose tissue and insulin resistance [2,3]. Virgin olive and extra virgin olive oils are the only non-refined oils widely consumed in the crude form worldwide. In the Mediterranean diet, the extra virgin oil is considered an important source of antioxidants and unsaturated fats which are associated with decreased risk of cardiovascular disease, metabolic syndrome, obesity, type-2 diabetes and hypertension [4,5]. 

With the exception of olive oil, most vegetable oils need to be refined because during the post-harvest handling, undesirable substances such as phospholipids, monoacylglycerols, diacylglycerols, free fatty acids, pigments, flavor components and sulfur compounds can be incorporated into the oils [6]. The oil refining process consists of degumming, neutralization, washing and drying steps, which improve some physicochemical characteristics such as acidity and the percentage of free fatty acids and increases thermal stability [7]. However, this process can result in degradation of sensitive bioactive compounds such as tocopherols [6] and carotenoids [7]. In this context, *in vitro* studies involving vegetable oils before and after refinement have been carried out to evaluate if processing compromises the functional potential of these oils [8,9,10]. 

Many studies have been performed to evaluate the effect of the consumption of vegetable oils on lipid metabolism and vitamins antioxidant status in animal models [11,12,13]. Although some *in vitro* studies have been conducted in order to highlight the benefits of the Brazilian palm (buriti) oil related to its lipid composition and quantification of vitamins A and E [7,14], there is a gap in knowledge related to the effects of the consumption of buriti oil in the diet on metabolism, particularly before and after the refining process. Buriti palm (*Mauritia flexuosa*) is a species native to Brazil and considered one of the most important vegetal sources of carotenoids and vitamin A [15]. Buriti oil is usually consumed as a crude oil presenting 3.1% saturated fatty acids, 92.3% monounsaturated fatty acids, 4.6% polyunsaturated fatty acids and considerable amounts of β-carotene (911.4 mg·kg^−1^) and tocopherol (800 mg/kg^−1^) [7,16]. In this context, the present study aimed to evaluate the influence of the addition of crude or refined buriti oil in the diets on the biochemical and murine parameters and vitamin A and E status in young rats. 

## 2. Results and Discussion 

### 2.1. Assessment of Diet Intake, Weight Gain and Feed Efficiency Coefficient (FEC) 

Weight gain and diet intake did not differ between groups (*p* > 0.05) receiving crude or refined buriti oil (Table 1). Similar results were reported by Berger *et al.* [17] in a study assessing the effects of addition of crude or refined amaranth oil (*Amaranthus caudatus* L.) in the diet of hamsters. 

**Table 1:** Intake, weight gain and feed efficiency coefficient of Wistar rats fed with diets added of soybean oil or refined or crude buriti oil.

Variables	Animal Groups		
	CG (n = 10)	CBO (n = 10)	RBO (n = 10 )
Mass gain (g)	104.98 ± 14.53 ^a^	91.37 ± 13.41 ^a^	110.36 ± 18.16 ^a^
Diet intake (g)	336.9 ± 36.72 ^a^	344.25 ± 25.67 ^a^	336.55 ± 21.04 ^a^
FEC	0.30 ± 0.07 ^ab^	0.26 ± 0.03 ^b^	0.34 ± 0.07 ^a^

Means followed by different letters in the same line indicate a significant difference error probability *p* < 0.05, according to the Tukey test. Abbreviations: FEC = feed efficiency coefficient. Groups: CG = control group with AIN-93G diet; CBO = experimental group with AIN-93G diet added of crude buriti oil; RBO = Experimental group with AIN-93G diet added of refined buriti oil.

However, the FEC was higher for group receiving diet with refined buriti oil (RBO) compared to that receiving crude buriti oil (CBO). This difference is probably related to the presence of hydrophilic substances such as glycerol, methanol and water, which are reduced during refinement and affect the nutritional value of oils and may also affect feed conversion [18]. 

### 2.2. Murine Anthropometric Parameters 

No differences were observed in the murine parameters of animals receiving buriti (CBO and RBO) and soybean oils (control group), except for abdominal circumference (AC), which was lower (*p* < 0.05) in the control group compared to the other groups (Table 2). 

**Table 2:** Murine variables of Wistar rats fed with diets added of soybean oil or refined or crude buriti oil.

Murine Antropometric Data	Animal Groups		
	CG (n = 10)	CBO (n = 10)	RBO (n = 10 )
TC (cm)	10.55 ± 1.23 ^a^	11.14 ± 0.45 ^a^	10.9 ± 0.46 ^a^
AC (cm)	10.86 ± 1.43 ^b^	12.41 ± 1.07 ^a^	12.25 ± 0.75 ^a^
AC/TC	1.03 ± 0.05 ^a^	1.11 ± 0.03 ^a^	1.12 ± 0.05 ^a^
BL (cm)	18.27 ± 1.42 ^a^	18.09 ± 1.04 ^a^	18.40 ± 0.97 ^a^
BW (g)	136.16 ± 31.38 ^a^	146.20 ± 20.09 ^a^	146.23 ± 24.05 ^a^
BMI (g·cm^−2^)	0.40 ± 0.05 ^a^	0.45 ± 0.04 ^a^	0.43 ± 0.04 ^a^
Lee index	0.28 ± 0.01 ^a^	0.29 ± 0.01 ^a^	0.26 ± 0.09 ^a^

Means followed by different letters in the same line indicate a significant difference error probability *p* < 0.05, according to the Tukey test. Abbreviations: CT = chest circumference; AC = abdominal circumference; BL = body length; BW = body weight; BMI = Body mass index. Groups: CG = control group with AIN-93G diet; CBO = experimental group with AIN-93G diet added of crude buriti oil; RBO = Experimental group with AIN-93G diet added of refined buriti oil.

However, the AC value observed in the control group is consistent with reports of rats fed with standard diet [19]. BMI, considered the most sensitive index to detect obesity and carcass fat [15] ranged from 0.40 to 0.45 g·cm^−2^ in the animals studied, those values are within normal parameters (0.38 and 0.68 g·cm^−2^) for animals aged 30–150 days. The BMI values observed in this study were lower than those reported by Santillan *et al.* [20] for rats fed diets with added soybean or sunflower oil (0:50 to 0:57). 

### 2.3. β-Carotene and Alpha Tocopherol in Diet, Blood and Liver of Rats 

**Figure 1:**
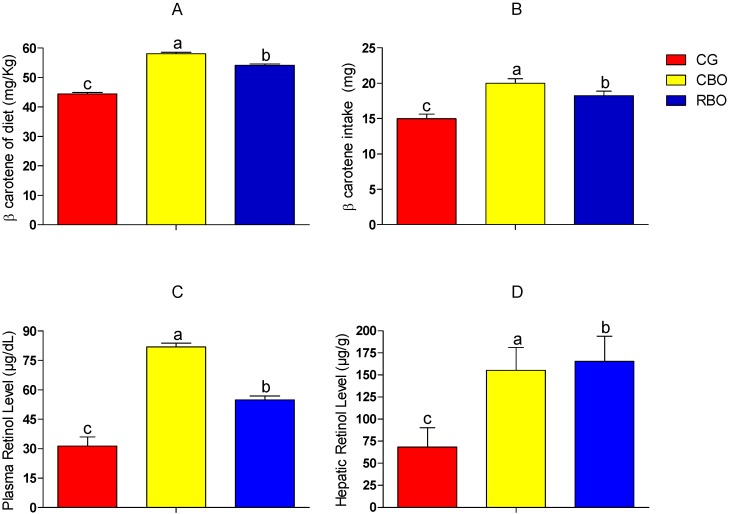
Content and intake of β-carotene of diet and serum and hepatic retinol of Wistar rats fed with diets added of soybean oil or refined or crude buriti oil. Panel **A**: β-carotene of diet; Panel **B**: β-carotene intake; Panel **C**: Plasma retinol level; Panel **D**: Hepatic retinol level. Means followed by different letters indicate significant difference with error probability *p* < 0.05, according to the Tukey test. Abbreviations: Groups: CG = control group with AIN-93G diet; CBO = experimental group with AIN-93G diet with crude buriti oil; RBO = Experimental group with AIN-93G diet with refined buriti oil.

Diet containing crude buriti oil had higher vitamin A content (104,652 IU/kg of diet equivalent to 58.14 mg β-carotene) compared to diet containing refined buriti oil (97,524 IU/kg of diet equivalent to 54.18 mg β-carotene). The lowest vitamin A concentration was found in diet added of soybean oil (80,028 IU/kg equivalent to 44.46 mg of β-carotene) (Figure 1). Diets added of crude or refined buriti oil had higher vitamin A content than values observed by Ramos *et al.* [21] and Siqueira *et al.* [22] for diets containing whole pulp of acrocomia (*Acrocomia aculeata*) and cassava (*Manihot esculenta crantz*), respectively.

FAO/WHO [23] and the Institute of Medicine [24] recommend that the daily uptake of retinol equivalent (RE) in the body be about 1.00 mg RE/day for men, 0.8 mg/day for women and 0.48 mg/day for children, which corresponds to 12 mg β-carotene/day for men, 9.6 mg β-carotene/day for women and 4.8 mg β-carotene/day for children, respectively, whereas 1 µg RE is equal to 12 µg β-carotene. Respecting the limited extrapolations of results of the present study for the human status, diet added of crude buriti oil supplies almost five times the recommended daily uptake of RE/day for men, six times daily uptake of RE/day for women and 12 times the recommended daily uptake of RE/day for children. On the other hand, refined buriti oil supplies 4.5 times the daily uptake of RE/day for men, almost 6 times the daily uptake of RE/day for women and 11 times the daily uptake of RE/day for children. 

The highest vitamin A concentration in plasma was found for the CBO group, confirming the loss of pigments and vitamins often reported for vegetable oils submitted to refinement [7,10]. Hepatic vitamin A levels did not differ between groups receiving buriti oil (*p* > 0.05), which were higher than those found for the control group (*p* < 0.05). Plasma retinol is homeostatically controlled, *i.e.*, plasma retinol concentration does not decrease until liver reserves are depleted, which explains the results found [22], suggesting that buriti oil is a major source of dietary vitamin A. 

Even though the group that consumed refined buriti oil added to the diet (RBO) ingested lower amounts of β-carotene, the accumulation of this compound in the liver was the same as the group that ingested greater amount of β-carotene or that consumed crude buriti oil added to the diet (CBO). The RBO group showed greater efficiency in the conversion of β-carotene from diet on the body reserves of animals. Comparing the serum and hepatic retinol of RBO group (54.88 µg/dL and 165.49 µg/g) with results obtained by Yuyama *et al.* [25] (35.21 µg/dL and 124.15 µg/g) for rats fed with lyophilized buriti pulp, it was observed that there was probably a more efficient conversion of β-carotene from diets containing buriti oil, which resulted in higher plasma and hepatic retinol concentrations. 

**Figure 2:**
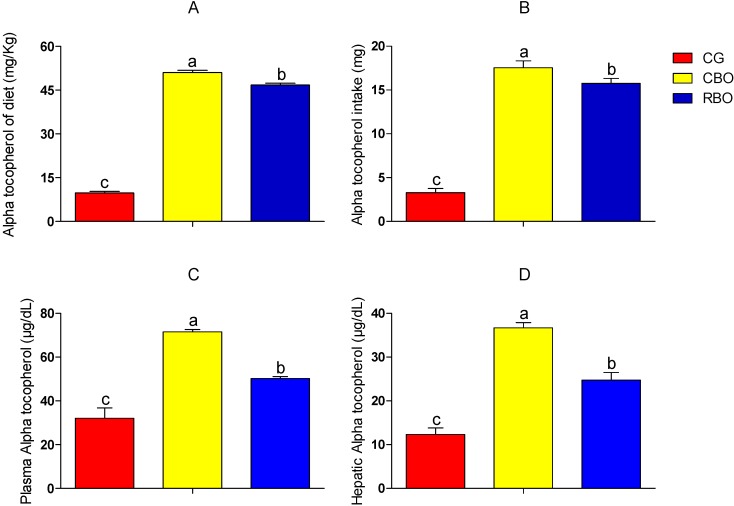
Content and intake of alpha tocopherol of diets and serum and hepatic alpha tocopherol of Wistar rats fed with diets added of soybean oil or refined or crude buriti oil. Panel **A**: alpha tocopherol of diet; Panel **B**: alpha tocopherol intake; Panel **C**: Plasma alpha tocopherol level; Panel **D**: Hepatic alpha tocopherol level. Means followed by different letters indicate significant difference with error probability *p* < 0.05, according to the Tukey test. Abbreviations: Groups: CG = control group with AIN-93G diet; CBO = experimental group with AIN-93G diet with crude buriti oil; RBO = Experimental group with AIN-93G diet with refined buriti oil.

Although the diet intake was similar among groups (*p* > 0.05), the serum and hepatic α-tocopherol concentration was higher in CBO and RBO groups, respectively, indicating the presence of this compound in greater amounts in diets added of buriti oil (Figure 2).The use of animal models allows simulating the event investigated in humans, thus, considering the data obtained, it is possible to calculate the efficiency of diet added of buriti oil in relation to guidelines of the Dietary Reference Intakes—DRIs [24] and vitamin contents E for humans. The DRI recommendation for vitamin E for adults is 10 mg/day and for children is on average 8.5 mg/day, and it was observed that the diet consumed by the CBO group (51.00 ± 0.82 mg of alpha tocopherol/kg) could correspond to supply almost five times the DRI value for adults and six times the DRI values for children, while the diet consumed by the RBO group (46.80 ± 0.60 mg of alpha tocopherol/kg) could supply almost five times the DRI values for adults and five and a half times the DRI values for children. Therefore, it could be inferred that diet added of crude or refined buriti oil is a source of vitamin E and is able to supply more than 15% of DRIs for adults and children. In addition, due to its antioxidant properties, tocopherol provides greater chemical stability to buriti oil, increasing its shelf life and minimizing biochemical oxidation reactions [26], which may contribute to the processing of this oil.

The difference between the amounts of serum tocopherol detected in animals fed with crude buriti oil (71.6 ± 1.00 µg/dL) compared with those who consumed refined buriti oil (50.2 ± 0.90 µg/dL) results from the reduction of this compound during the refining process [10]. However, both groups had serum tocopherol contents higher than those found by Dromitrovic *et al.* [27] in animals fed with olive oil or corn oil, 29 μg/dL and 33 μg/dL, respectively. The results of the present study also showed higher serum vitamin E concentrations in rats fed with coconut oil (14.9 ± 2.26 µmol/mmol) or hydrogenated vegetable fat (8.67 ± 1.61 µmol/mmol) found by Naziroglu and Brandsch [28]. 

The liver α-tocopherol concentration in the CBO group (36.68 ± 1.19 µg/g) was three times higher and that of the RBO group (24.7 ± 1.79 µg/g), and twice (*p* < 0.05) the α-tocopherol concentration found in the liver of animals fed with soybean oil (12.33 ± 1.5 µg/g), which demonstrates the easy absorption of vitamin E from buriti oil. 

The amount of vitamin E found in the liver of rats fed with diet added of crude buriti oil (CBO) was higher than that reported for rats consuming canola oil and olive oil, 25 μg/g and 33 μg/g [27]. In addition, CBO and RBO groups showed liver tocopherol concentrations higher than those found by Naziroglu and Brandsch [28] in the liver of rats that consumed coconut oil (19.8 ± 10.3 µg/g) and hydrogenated vegetable fat (19.6 ± 9.8 µg/g). 

Studies have reported that the consumption of oils containing high concentration of natural antioxidants such as tocopherol may protect against possible physiological damage such as lipid peroxidation, play a role in the healing process and improve the antioxidant defense system [27,29,30,31,32]. 

The diets offered to the animals in this study simulated the daily consumption of soybean oil, crude oil or refined buriti without interference from the consumption of other vegetable oils, in order to evaluate and compare the individual effects of each oil in an animal model. However, it is important to highlight that the amounts of carotenoids and tocopherols present in diets compared with the intake recommendations of these nutrients for humans [23,24] should be evaluated with caution, respecting the experimental design of the study, the substance tested of the dose translated from animal to humans and the time window used to test the hypothesis [32,33,34]. All these factors must consider the “R” of refinement and the “R” reduction that are essential in animal studies and must respect the limitations in translating the animal model used, not just as a simple conversion of results obtained in animal models to humans [32,33,34].

### 2.4. Lipid and Haematological Profiles 

Regarding data on red blood cells, there was a higher percentage of hematocrit, *i.e.*, greater amount of red blood cells in the group receiving diet added of crude buriti oil when compared to the other groups, but no difference was found in the hemoglobin concentration (Table 3). 

**Table 3:** Lipid and haematological profile of Wistar rats fed with diets added of soybean oil or refined or crude buriti oil.

Biochemical Data	Animals Groups		
	CG (n = 10)	CBO (n = 10)	RBO (n = 10 )
Hemoglobin (g/dL)	10.55 ± 1.23 ^a^	11.14 ± 0.45 ^a^	10.9 ± 0.46 ^a^
Hematocrit (%)	41.38 ± 8.4 ^ab^	48.25 ± 3.11 ^a^	35.25 ± 10.24 ^b^
TC (mg/dL)	96.83 ± 13.97 ^b^	131.51 ± 15.52 ^a^	52.25 ± 6.88 ^c^
HDL-C (mg/dL)	24.38 ± 11.6 ^a^	23.64 ± 2.04 ^a^	13.41 ± 1.59 ^b^
TC/HDL-C (mg/dL)	3.91 ± 0.64 ^b^	5.44 ± 0.46 ^a^	3.96 ± 0.31 ^b^
VLDL-C (mg/dL)	16.18 ± 6.36 ^a^	19.88 ± 2.35 ^a^	8.86 ± 0.86 ^b^
LDL-C (mg/dL)	57.88 ± 3.86 ^b^	87.49 ± 5.28 ^a^	30.84 ± 5.24 ^c^
TAG (mg/dL)	81.50 ± 32.33 ^a^	99.38 ± 11.75 ^a^	44.25 ± 4.68 ^b^
AST (IU/L)	10.75 ± 1.58 ^ab^	12.75 ± 1.91 ^a^	10.00 ± 2.78 ^b^
ALT (IU/L)	12.13 ± 1.81 ^a^	14.38 ± 2.00 ^a^	11.75 ± 2.55 ^a^

Means followed by different letters in the same line indicate a significant difference error probability *p* < 0.05, according to the Tukey test. Abbreviations: triacylglycerol (TAG); Serum cholesterol (TC); high density lipoprotein cholesterol (HDL-C); low density lipoprotein cholesterol (LDL-C); VLDL = very low density lipoprotein cholesterol; ALT = alanine aminotransferase; AST = aspartate aminotransferase. Groups: CG = control group with AIN-93G diet; CBO = experimental group with AIN-93G diet with crude buriti oil; RBO = Experimental group with AIN-93G diet with refined buriti oil

The consumption of β-carotene increases iron absorption in the human body [35], which is in agreement with results found, in which the highest hematocrit percentage was observed in the group that ingested higher vitamin A contents, that is, animals that received crude buriti oil (CBO). 

The results observed for HDL-C, LDL-C and TC in the group of animals that received refined buriti oil (RBO) are in agreement with those obtained by Asadi *et al.* [11] for rats fed with diet containing corn oil (HDL-C 16.75 mg/dL, LDL-C 11.28 mg/dL and TC 45.03 mg/dL), grape seed oil (HDL-C 14.59 mg/dL, LDL-C 28.06 mg/dL and TC 59.60 mg/dL) and canola oil (HDL-C 18.26 mg/dL LDL-C 22.86 mg/dL and TC 55.81 mg/dL). TAG, TC and HDL-C concentrations in groups fed with diet added of crude (CBO) or refined buriti oil (RBO) were lower than those found by Tzang *et al.* [36] in hamsters fed with crude linseed oil (TAG 100-200 mg / dL, TC 177.59 mg/dL and HDL-C approx. 70 mg/dL). TAG and TC concentrations in group fed with diet added refined buriti oil (RBO) were similar those founds by Quiles *et al.* [37] in rats fed with diet containing olive oil (TAG 56.7 mg/dL, TC 68.8 mg/dL). It is also important to emphasize that despite the consumption of buriti oil have shown beneficial health effects, the consumption of olive oil should be highlighted because it is a non-atherogenic oil among all the oils consumed in the world and present an excellent profile fatty acids, a high content of antioxidants such as polyphenols and fat-soluble vitamins [38,39]. Arbonés-Mainar *et al.* [40] showed that consumption of a Western diet supplemented with one of several varieties of extra virgin olive oil decreased atherosclerosis lesions, reduced plaque size, and decreased macrophage recruitment in animal model. The effects of olive oil consumption [27,30,37,40] and various other oils such as soybean [28,41], corn, canola [11,30,42], among others [12,17,36,42], have already been evaluated and were well documented in animal models and translating these results to humans [4,43,44], with similar effects among models, respecting the extrapolation of results.

There was a decrease in all serum lipid parameters analyzed of animals fed with diet containing refined oil buriti (RBO) compared to those fed with diet added of crude buriti oil (CBO). This result agrees with results obtained by Berger *et al.* [17], who compared the blood lipid profile of rats fed with crude and refined amaranth oil. However, higher total cholesterol values were observed for group receiving diet added of crude buriti oil (CBO). This result suggests that although crude oils have higher concentration of phytosterols, vitamins, antioxidants and pigments [6], which are associated with reduced serum cholesterol and oxidative stress, crude oils also have higher concentration of oxidized substances and impurities such as peroxides, hydroperoxides, volatile (aldehydes, ketones) and nonvolatile compounds (carbonyls and cyclic fatty acids), which have been associated with dyslipidemia, hypertension, inflammation, oxidative stress, endothelial dysfunction and atherosclerosis [45,46]. 

The refining process removes oxidized substances; thus, even if the process causes loss of approximately 13.08% of β-carotene and 12.46% of tocopherol (compared to the crude buriti oil), the removal of the oxidized substances might be related to the greater effectiveness of refined Buriti oil in lowering LDL-C and total cholesterol levels in rats from the RBO group. Probably, although carotenoids and tocopherols were reduced during the refining process, the quantities of antioxidants present in refined buriti oil were effective to minimize lipoprotein oxidation and consequently reduce the plasma levels of animals (RBO) [47,48]. 

The consumption of refined buriti oil reduces the concentration of triglycerides, total cholesterol and fractions (HDL-C, LDL-C and VLDL-C) in healthy rats. These results are relevant but should be carefully assessed with regard to the consumption of refined buriti oil associated with the prevention and treatment of cardiovascular diseases and dyslipidemias, considering that the animal model used in this study did not perform the experiment with dyslipidemia-induced rats.

Serum aminotransferase levels (ALT and AST) are reliable indicators of functional or structural alteration of liver cell [49]. In the present study, the ALT values obtained ranged from 11.75 to 14.38 IU and AST values from 10.00 to 12.75 IU, which are lower than those observed by Ohara *et al.* [42] in rats fed with diets added of rapeseed oil (18.1 IU ALT and 59.8 IU AST). The CBO group showed the highest AST value; however, the ALT concentration showed no difference among groups (*p* < 0.05). According to Sugiura *et al.* [50], there is an inverse association of serum retinol concentrations with serum AST and ALT, which was not observed in this study. However, according to Yeh *et al.* [46], ALT and AST aminotransferases can increase with increasing consumption of oxidized oil, suggesting that the higher AST values found in CBO group may be related to the presence of oxidation products, which would be removed during the refining process of oils.

## 3. Experimental Section 

### 3.1. Oil Samples 

The oils used in this study were all obtained from six different batches mixed to form the test sample. Buriti oil samples were obtained and characterized as Aquino *et al.* [7]. The buriti oil was extracted by hand from ripe fruit by cooking in water for 20 min at temperature of ±60 °C and the oil was separated from the aqueous fraction. Subsequently, crude oil was used in the refining process, followed by degumming, neutralization, washing and drying steps [7]. The refined soybean oil (Soya^®^, São Paulo, Brazil) was purchased in supermarket of João Pessoa, Brazil, was used as control and characterized according previously described procedures AOAC [51]. The composition of soybean oil (control), as well as crude and refined buriti oil was analyzed in triplicate (Table 4).

**Table 4:** Fatty acids, β-carotene and tocopherol present in soybean oil, crude and refined buriti oils.

Composition	Vegetable Oils		
	Soybean Oil *	Crude Buriti Oil	Refined Buriti Oil
β-Carotene (mg·kg^−1^)	420.02 ± 2.80 ^c^	911.40 ± 2.40 ^a^	792.10 ± 4.54 ^b^**
Tocopherol (mg·kg^−1^)	305.20 ± 2.10 ^c^	810.00 ± 2.70 ^a^	709.00 ± 2.40 ^b^***
**Fatty Acids (%)**			
Miristic acid-C 14:0	17.10 ± 0.02 ^a^	0.50 ± 0.04 ^b^	0.50 ± 0.02 ^c^
Margaric acid-C 17:0	11.20 ± 0.03 ^b^	0.30 ± 0.02 ^a^	0.20 ± 0.01 ^c^
Stearic acid-C18:0	3.40 ± 0.03 ^a^	2.30 ± 0.02 ^b^	3.90± 0.01 ^c^
**Total saturated fatty acids—SFA%**	**31.70 ± 0.08 ^a^**	**3.10 ± 0.08 ^c^**	**4.6 ± 0.04 ^a^**
Palmitoleic acid-C16:1	n.d. ****	19.60 ± 0.02	19.40 ± 0.01
Oleic acid-C18:1	23.60 ± 0,04 ^b^	72.70 ± 0.02 ^a^	72.20 ± 0.02 ^c^
**Total monounsaturated fatty acids—MUFA%**	**23.60 ± 0.04 ^b^**	**92.30 ± 0.04 ^a^**	**91.60 ± 0.03 ^c^**
Linoleic acid-C 18:2	39.90 ± 0.02 ^a^	2.60 ± 0.04 ^b^	2.30 ± 0.01 ^c^
Linolenic acid-C 18:3	4.80 ± 0.02 ^a^	2.00 ± 0.01 ^b^	1.50 ± 0.01 ^c^
**Total polyunsaturated fatty acids—PUFA%**	**44.70 ± 0.04 ª**	**4.60 ± 0.05 ^b^**	**3.80 ± 0.01 ^c^**

Means followed by different letters in the same line indicate significant difference error probability *p* < 0.05, according to the Tukey test. * Control oil; ** Loss of β-carotene after refining = 13.08%; *** Tocopherol loss after refining = 12.46%; **** n.d. = not detected.

### 3.2. Animals and Diet 

The experimental protocol was approved by the Ethics Committee for Animal Research—CEPA—UFPE under number 23076.015472/2009-25 and followed the guidelines of the Brazilian College for Animal Experimentation—COBEA. Thirty just-weaned male Wistar rats aged ±21 days coming from the Animal facilities of the Department of Nutrition, Federal University of Pernambuco (UFPE) were used. The animals were randomized into three groups, each with 10 animals and kept in individual cages with *ad libitum* water and diet, temperature of 22 ± 1 °C, relative humidity between 50% and 55%, and dark/light cycle of 12 h. 

The levels of macro and micronutrients in diets were calculated and balanced following recommendations of the American Institute of Nutrition (AIN) [52]. The level of oils (soybean, refined or crude buriti oils) added in diets was defined as 7 g oil/100 g diet for growing rodents (AIN-93 G), in accordance with the rules of American Institute of Nutrition (AIN) [52]. Considering the results observed in preliminary assays and results already published [19,20] about the influence of oil consumption on the lipid biochemical and metabolism of fat-soluble vitamins, the diets containing soybean, refined or crude buriti oils were administered for 28 days from weaning. The control group (CG) received diet containing soybean oil as lipid source; one experimental group received diet containing crude buriti oil (CBO) as lipid source and the other experimental group received diet containing refined buriti oil (BRO) as lipid source (Table 5). 

**Table 5:** Composition of diets of control group added of soybean oil (CG) and experimental groups added of crude (CBO) or refined buriti oil (RBO).

Ingredients *	Amounts (g/100 g)	CS	CBO	RBO
		Energy (Kcal)	Energy(Kcal)	Energy(Kcal)
Corn starch	52.9	186.10	186.10	186.10
Casein	20.0	68.00	68.0	68.0
Sucrose	10.0	40.00	40.00	40.0
Fiber	5.0	-	-	-
Soybean oil or buriti oil	7.0	63.0	63.0	63.0
Mix of minerals	3.5	-	-	-
Mix of vitamins	1.0	-	-	-
D.L-Methionine	0.3	-	-	-
Choline Bitartrate	0.3	-	-	-
Total	100	357.10	357.10	357.10

* AIN-93G diet [52].

On the basic diet of all groups, a mix of vitamins containing 4,000 IU of vitamin A (2.4 mg of β-carotene) and 75 IU of vitamin E (49.95 mg of α-tocopherol) were added to each kg of diet, as recommended by the American Institute of Nutrition (AIN) [18]. Weight of animals and diet consumption were weekly measured throughout the 28 days of the experimental period to determine the feed efficiency coefficient according to protocol proposed by Campbell [53]. 

### 3.3. Murine Anthropometric Parameters 

Murine parameters were obtained from anesthetized animals prior to euthanasia, and using a measuring tape, the following were measured: abdominal circumference (AC) immediately prior to the hind leg and chest circumference (CC), immediately behind the front leg, plus body weight and body length, measured from nose to base of tail. The Body Mass Index (BMI) was calculated by dividing body weight (g) by the squared length (cm^2^) and the Lee index (LI) was calculated by the cube root of body weight (g) divided by length (cm) [19]. 

After 28 days of experiment and after 12 h fasting, the animals were anesthetized with 1 mL ketamine hydrochloride and 1 ml xylazine hydrochloride per kg body weight. Heparin (500 IU/kg body weight) was intraperitoneally administrated as anticoagulant and the animals were perfused through the left ventricle (cardiac puncture) and the serum was used in biochemical analyses. The liver was removed for determination of vitamins A and E, washed with NaCl solution (0.9 g/100 mL), dried on absorbent paper and frozen until moment of analyses. 

### 3.4. Determination of Vitamins A and E in Diets and Intake of β-Carotene and Alpha Tocopherol by Rats 

Vitamins A and E were determined according to Prado *et al.* [54]. Briefly, each sample (5 g) was macerated and extracted with ethanol (10 mL) containing 0.25% butylated hydroxytoluene (BHT), distilled water (5 mL), and sodium chloride (0.5 g). Subsequently, hexane (10 mL) were added and the material was stirred and submitted to ultrasonic bath for 3 min for homogenization. After centrifugation (185× *g*/3 min/10 °C), the hexane extract was collected in amber glass. The procedure was performed five times. The hexane fraction was evaporated using nitrogen gas and after the removal of hexane, fractions were resuspended with 3.0 mL methanol and filtered through a 0.5-μm Millipore Fluoropore membrane. Filtered extract samples were injected into a High Performance Liquid Chromatography (HPLC) system (model 2699, Varian, Harbor City, CA, USA) coupled to a diode array detector (DAD) and a C18 chromatography column (Inertisil, 150 × 4.6 mm 5 μm, Chrompack-Varian, Middelburg, The Netherlands). Elution was performed with methanol: water (98:2) at flow rate of 1.0 mL·min^−1^. The injection volume was 20 μL, and detection wavelength of 325 nm. Sample identification was confirmed by comparing retention times to standard compounds under the same chromatographic conditions. Quantitation was accomplished by internal calibration using β-carotene (C-9750, Sigma, St. Louis, MO, USA) and α-tocopherol (T-3251, Sigma) standards. The intake of vitamin A as β-carotene and vitamin E as α-tocopherol were calculated as the amount of β-carotene and α-tocopherol, respectively, determined in the three types of diet based on of the amount of diet consumed by each group of animals. 

### 3.5. Determination of Serum and Hepatic Retinol and Alpha Tocopherol

Hepatic and serum retinol and α-tocopherol concentrations were determined by HPLC (model Ultimate 3000, Dionex, São Paulo, Brazil), using a C_18_ column measuring 4.6 × 2.50 mm × 5 µm, pre-column, detector set at 325 nm with methanol as mobile phase, flow rate of 1.5 mL·min^−1^ and peak holding at 3.6 minutes. For quantification of serum retinol and alpha tocopherol, 2 ml of blood were centrifuged (1665× *g*/10 min/10 °C) to remove the serum. Subsequently, 100 μL of sample was added to 100 μL of ethanol to precipitate proteins with stirring for 10 seconds. Then, 200 μL of hexane were added and stirred for 45 seconds and the material was centrifuged (1665× *g*/5 min/10 °C). Following this procedure, 100 μL of the supernatant were collected and submitted to evaporation with nitrogen [55]. The samples were dissolved again with 100 µL of methanol, from which 20 µL were taken to carry out chromatographic analyses. 

Hepatic retinol was quantified according to procedure adapted from Stahl *et al.* [56]. A total of 1 g of liver was added to ethanol (2 mL) and homogenized for 2 min using mechanical stirrer and then in vortex for another 2 min with the addition of hexane (2 mL). Subsequently, the material was centrifuged (18,501× *g*/10 min/10 °C), with drying of the supernatant under nitrogen atmosphere and resuspended in methanol (100 µL), from which 20 µL were taken to carry out chromatographic analyses. Quantitation was accomplished by internal calibration using retinol (R-7632, Sigma) and α-tocopherol (T-3251, Sigma) standards.

### 3.6. Lipid and Haematological Profiles 

Blood was collected from anesthetized animal by direct cardiac puncture (4 mL) and centrifuged (807× *g*/10 min/20 °C). Serum was kept at room temperature (25) for determination of cholesterol (enzyme) HDL-C (polyethylene glycol-PEG), LDL-C (polyethylene glycol-PEG), VLDL-C (polyethylene glycol-PEG) and triglyceride (enzymatic) using Dolles (Goiânia, GO, Brasil) kits according to manufacturer's instructions, as well as measurements of hematocrit and hemoglobin, aspartate transaminase (AST) and alanine transaminase (ALT). 

### 3.7. Statistical Analyses 

Randomized design was used and the analysis of results was performed using SPSS for Windows Evaluation Edition-14.0 (SPSS. Inc., Chicago, IL, USA) [57], considering the error probability (p) less than or equal (≤) to 5%. In order to meet the methodological assumptions of parametric tests and obtain consistent results, the sample homogeneity test was applied. The values obtained from three or more independent variables were submitted to analysis of variance (ANOVA) using the F test; however, for those with no homogeneity among samples, the Welch test for robustness was used, and for those showing significance, the Tukey’s test for multiple comparisons was applied.

## 4. Conclusions

The results obtained demonstrate for the first time the metabolic efficiency of crude or refined buriti oil used in the diet as a source of fatty acids and the antioxidant vitamins A and E. However, the consumption of refined buriti oil allows better feed conversion and improves the lipid profile by reducing LDL-cholesterol, triglycerides (TAG) and aspartate transaminase (AST), without changing the murine parameters of healthy rats.

## Abbreviations

AC: Abdominal circumference
AIN: American Institute of Nutrition
ALT: Alanine transaminase
AST: Aspartate transaminase
BMI: Body Mass Index
BHT: Butylated Hydroxytoluene
CBO: Experimental group that consumed diet added of casein and crude buriti oil
CG: Control group that consumed diet added of casein and soybean oil
CC: Chest circumference
CEPA: Ethics Committee on Animal Research
COBEA: Brazilian College of Animal Experimentation
DAD: Diode Array Detector
DRIs: Dietary Reference Intakes
FEC: Feed Efficiency Coefficient
HDL-C: High density lipoprotein
HPLC: High Performance Liquid Chromatography
IU: International Unit (vitamins)
LI: Lee index
LDL-C: Low density lipoprotein
RBO: Experimental group that consumed diet containing casein and refined buriti oil
RE: Retinol equivalents
TAG: triglycerides
UFPE: Federal University of Pernambuco
VLDL-C: Very low density lipoprotein
